# Supplementary figures and images for: DNA methyltransferase CHROMOMETHYLASE3 prevents ONSEN transposon silencing under heat stress
Source: PLoS Genet. 2021 Aug 19;17(8):e1009710. doi: 10.1371/journal.pgen.1009710 (PMC8376061; doi:10.1371/journal.pgen.1009710)

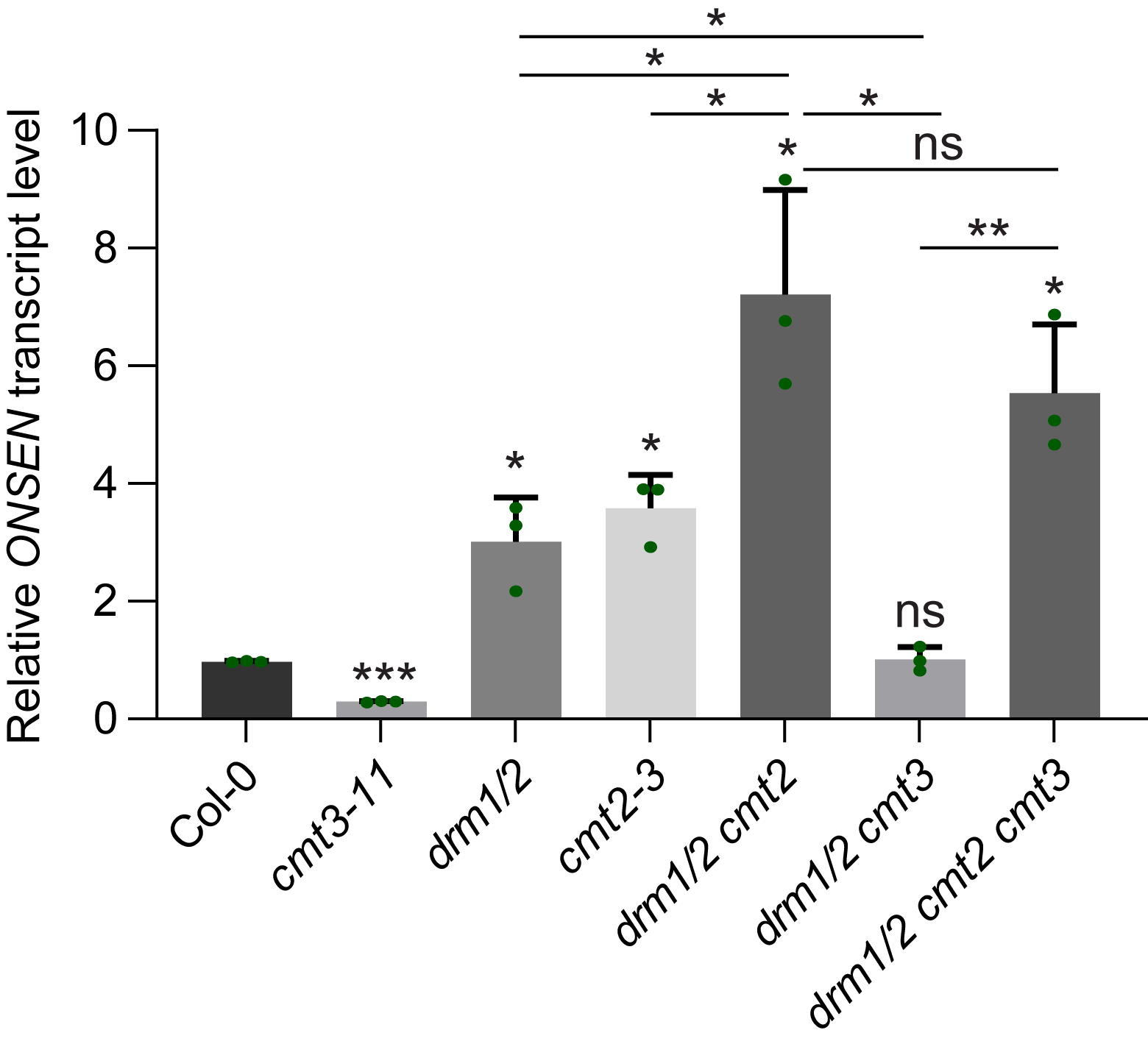

Supplement: S1 Fig — RT-qPCR showing relative ONSEN transcript level in the corresponding mutants under heat stress. All bars represent mean + SD from three biological replicates. The relative ONSEN transcripts were first normalized to 18S rRNA, and then to Col-0. Student’s t-test, *P < 0.05; **P < 0.01; ***P < 0.001. ns indicates not significant. (TIF) [file pgen.1009710.s001.tif]

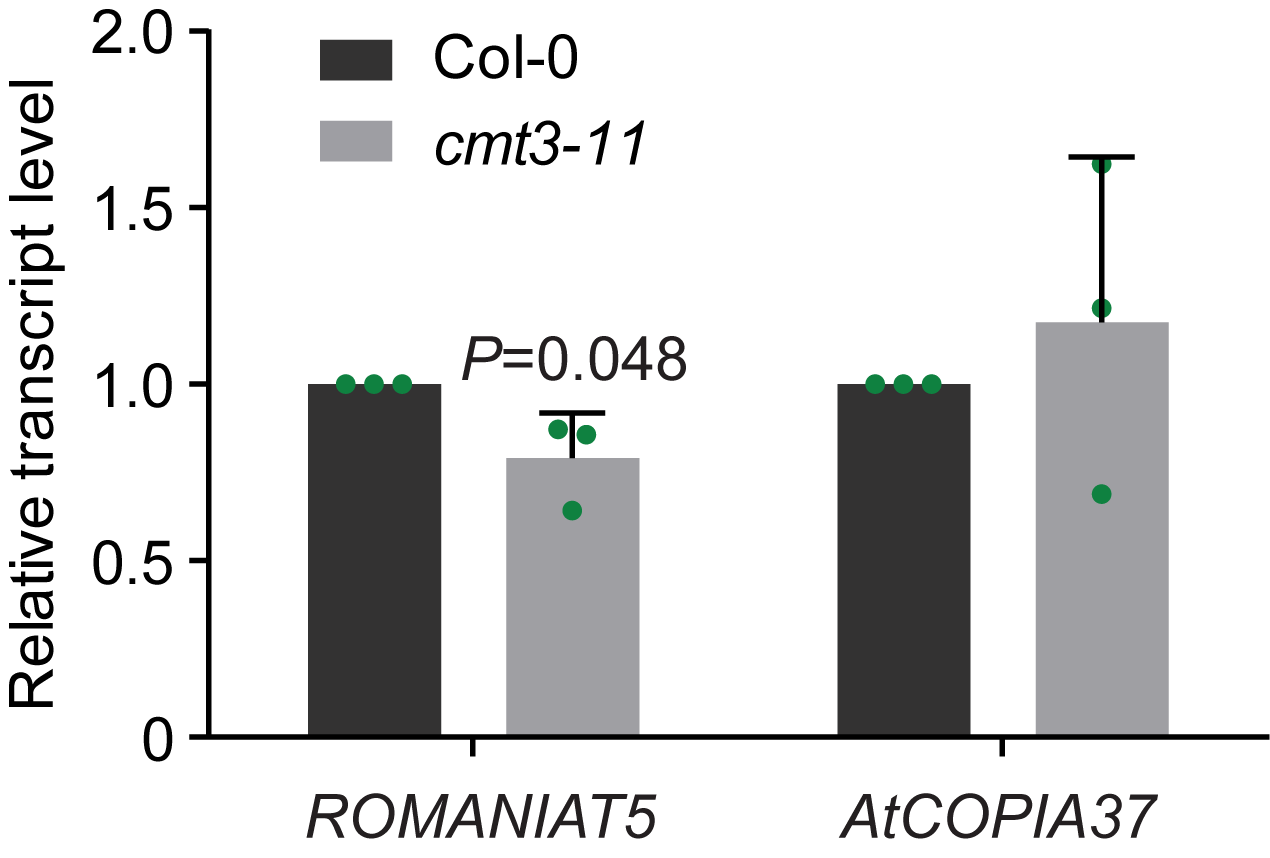

Supplement: S2 Fig — The relative transcript levels were determined by RT-qPCR, first normalized to ACT7, and then to Col-0. All bars represent mean + SD from three biological replicates. P value was determined by Student’s t-test. (TIF) [file pgen.1009710.s002.tif]

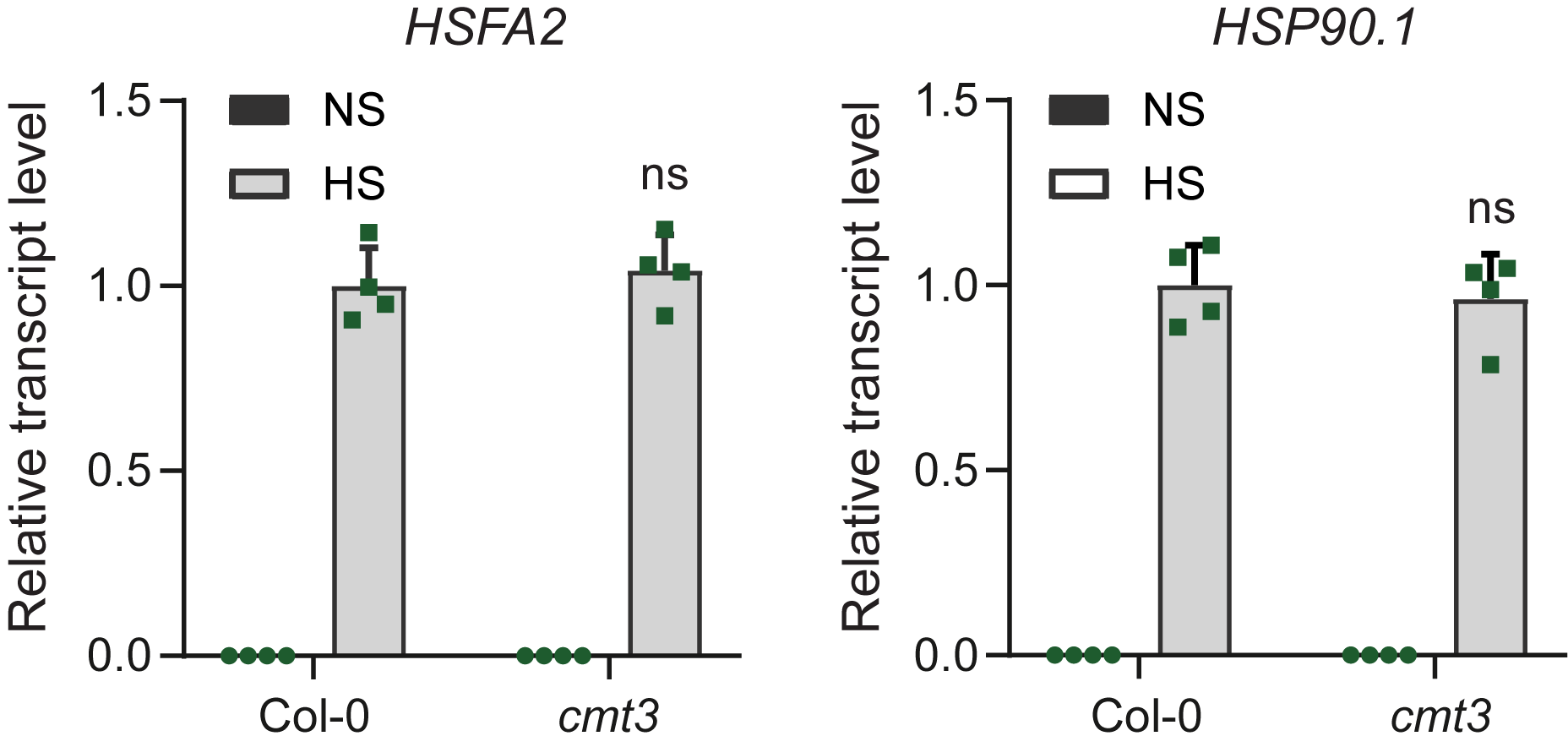

Supplement: S3 Fig — The transcripts were detected by RT-qPCR and normalized to 18S rRNA. All bars represent mean + SD from four biological replicates. Student’s t-test, ns indicates not significant. NS, non-stress; HS, heat stress. (TIF) [file pgen.1009710.s003.tif]

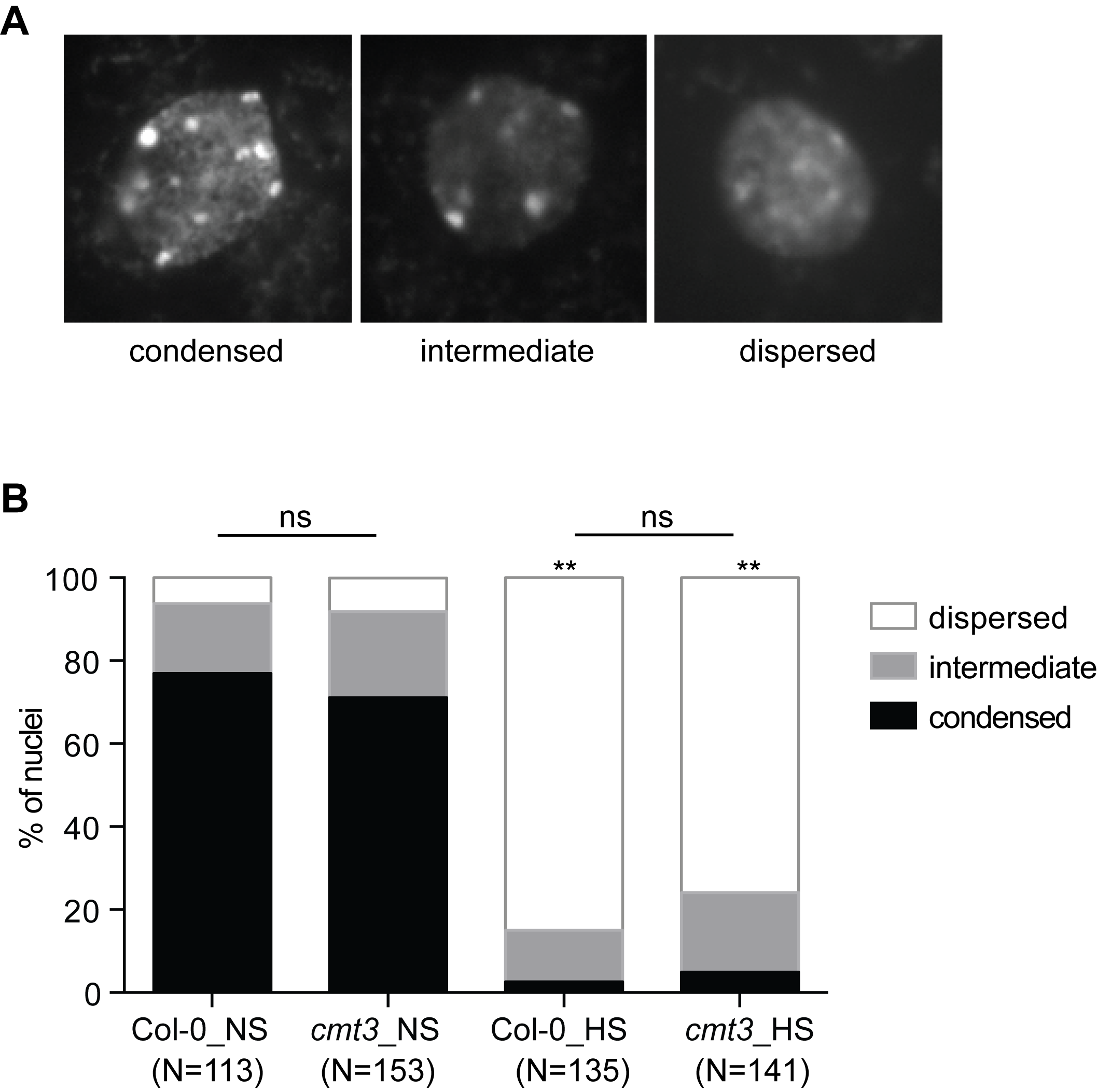

Supplement: S4 Fig — (A) Representative images of nuclei showing condensed, intermediate, and dispersed chromocenters by DAPI staining. (B) Bar graph indicating the proportion of nuclei displaying condensed, intermediate, and dispersed chromocenters in cmt3 and Col-0. N indicates the total number of nuclei counted. NS, non-stress; HS, heat stress. ** P < 0.01 by Chi-square test. ns indicates not significant. (TIF) [file pgen.1009710.s004.tif]

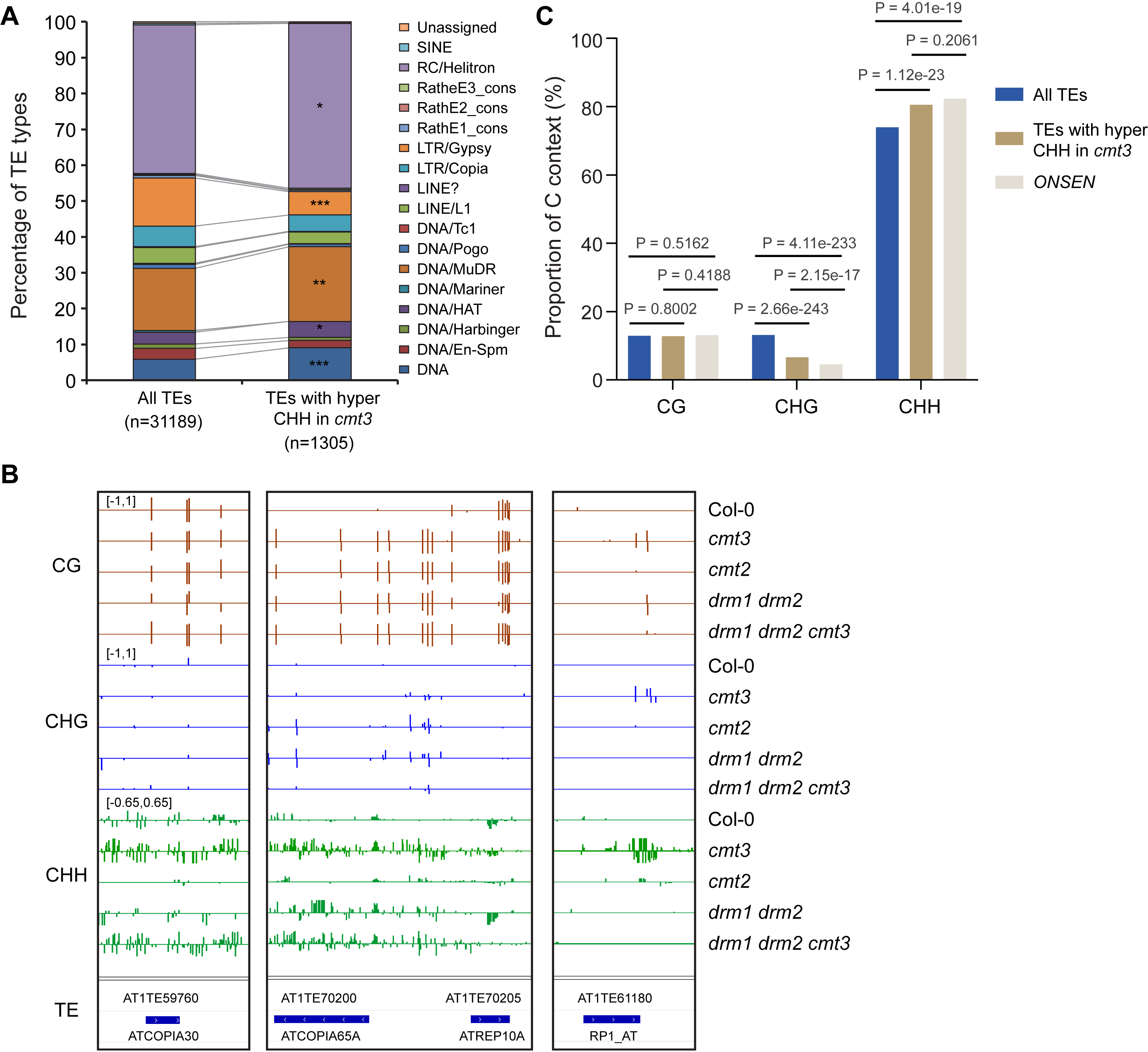

Supplement: S5 Fig — (A) Bar charts showing the proportion of each transposable element (TE) family in the genome that contains cmt3 hyper CHH differentially methylated regions. The P values were calculated using Fisher’s exact test, *P < 0.05, **P < 0.01, ***P < 0.001. (B) Genome browser snapshots of TEs showing hyper CHH methylation in cmt3. (C) The percentage of C context (CG, CHG, CHH) in ONSEN and other TEs with hyper CHH methylation in cmt3. The P values were calculated using Fisher’s exact test. (TIF) [file pgen.1009710.s005.tif]

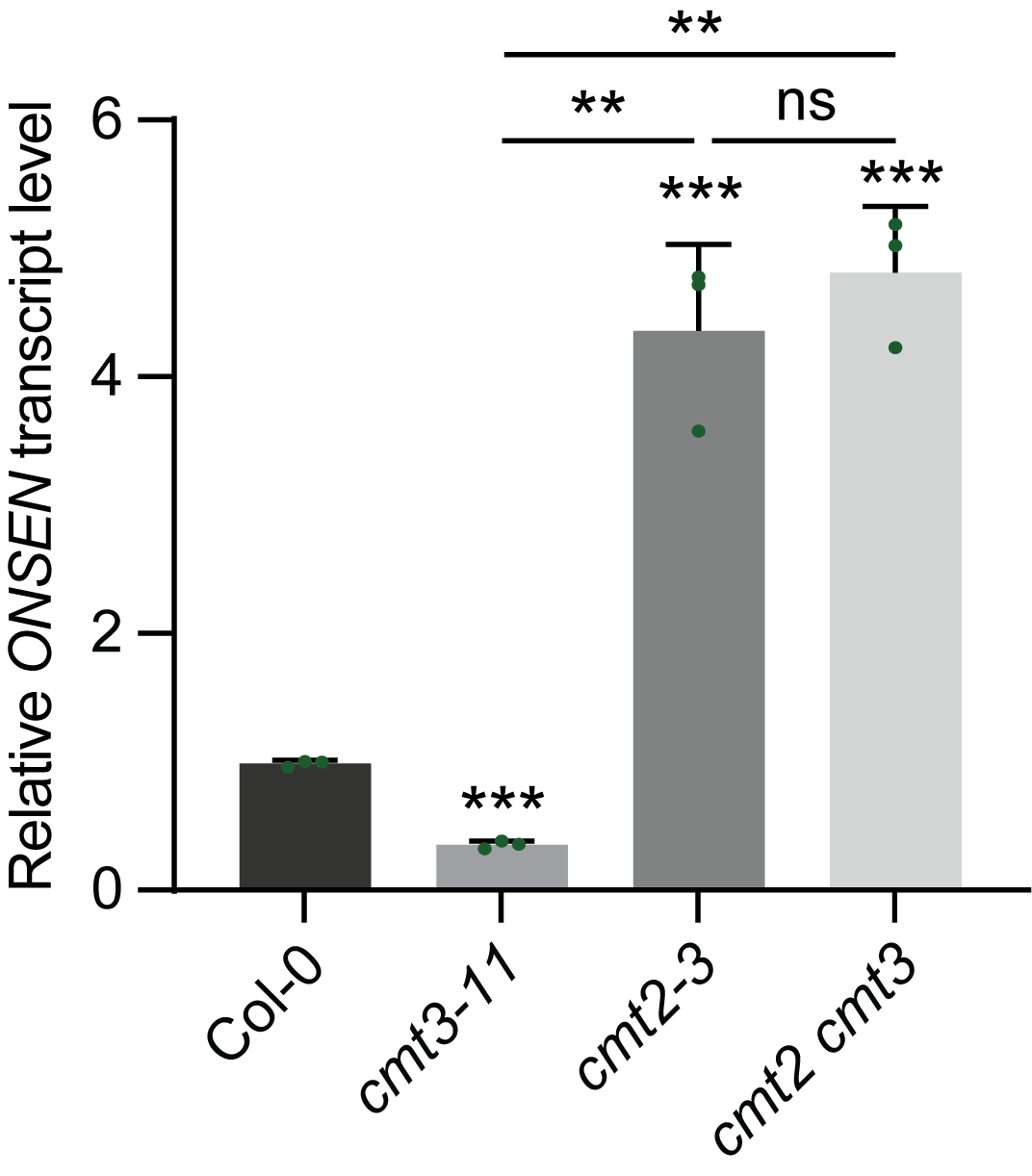

Supplement: S6 Fig — RT-qPCR showing the relative ONSEN transcript level in Col-0, cmt3-11, cmt2-3, and cmt2 cmt3 under heat stress. All bars represent mean + SD from three biological replicates. The ONSEN transcripts were first normalized to ACT7, and then to Col-0. Student’s t-test, **P < 0.01;***P < 0.001. ns indicates not significant. (TIF) [file pgen.1009710.s006.tif]

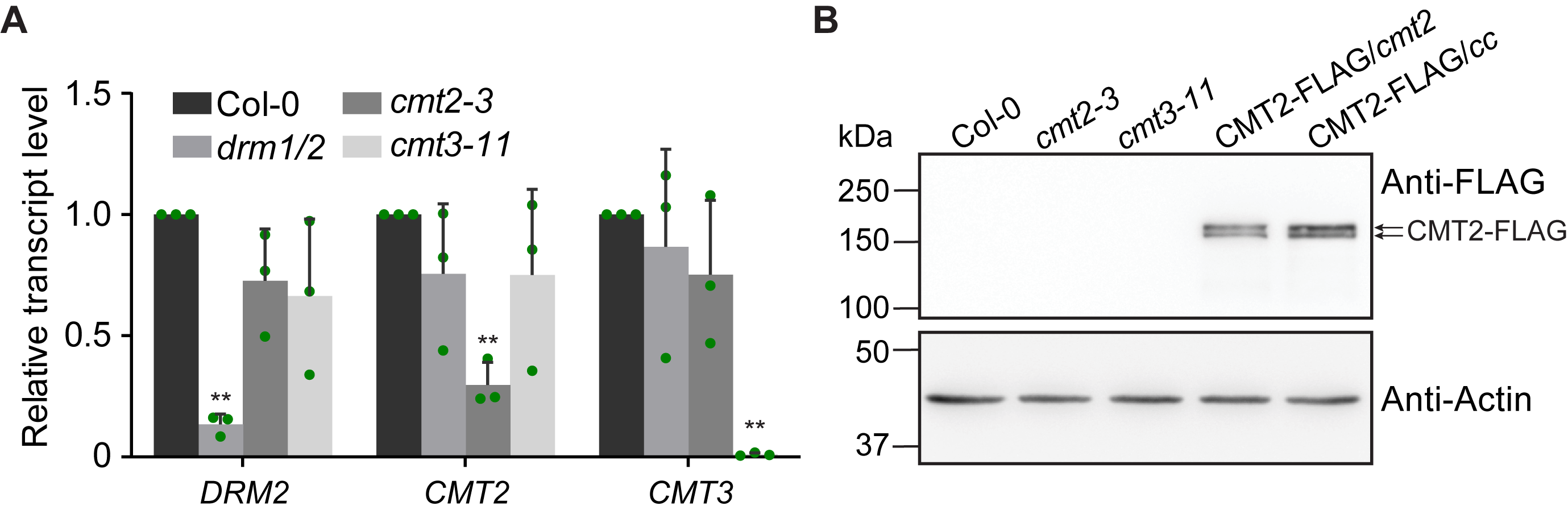

Supplement: S7 Fig — (A) RT-qPCR showing relative transcript levels of DRM2, CMT2, and CMT3 in Col-0, drm1 drm2 (drm1/2), cmt2-3, and cmt3-11 under heat stress. The relative transcripts were first normalized to ACT7, then to Col-0. Data are mean + SD from three biological replicates. Student’s t-test, **P < 0.01. (B) Immunoblot analysis of CMT2 protein in CMT2 tagged lines in cmt2 (CMT2-FLAG/cmt2) and cmt2 cmt3 background (CMT2-FLAG/cc). Arrows indicate CMT2-FLAG protein. Actin serves as a loading control. (TIF) [file pgen.1009710.s007.tif]

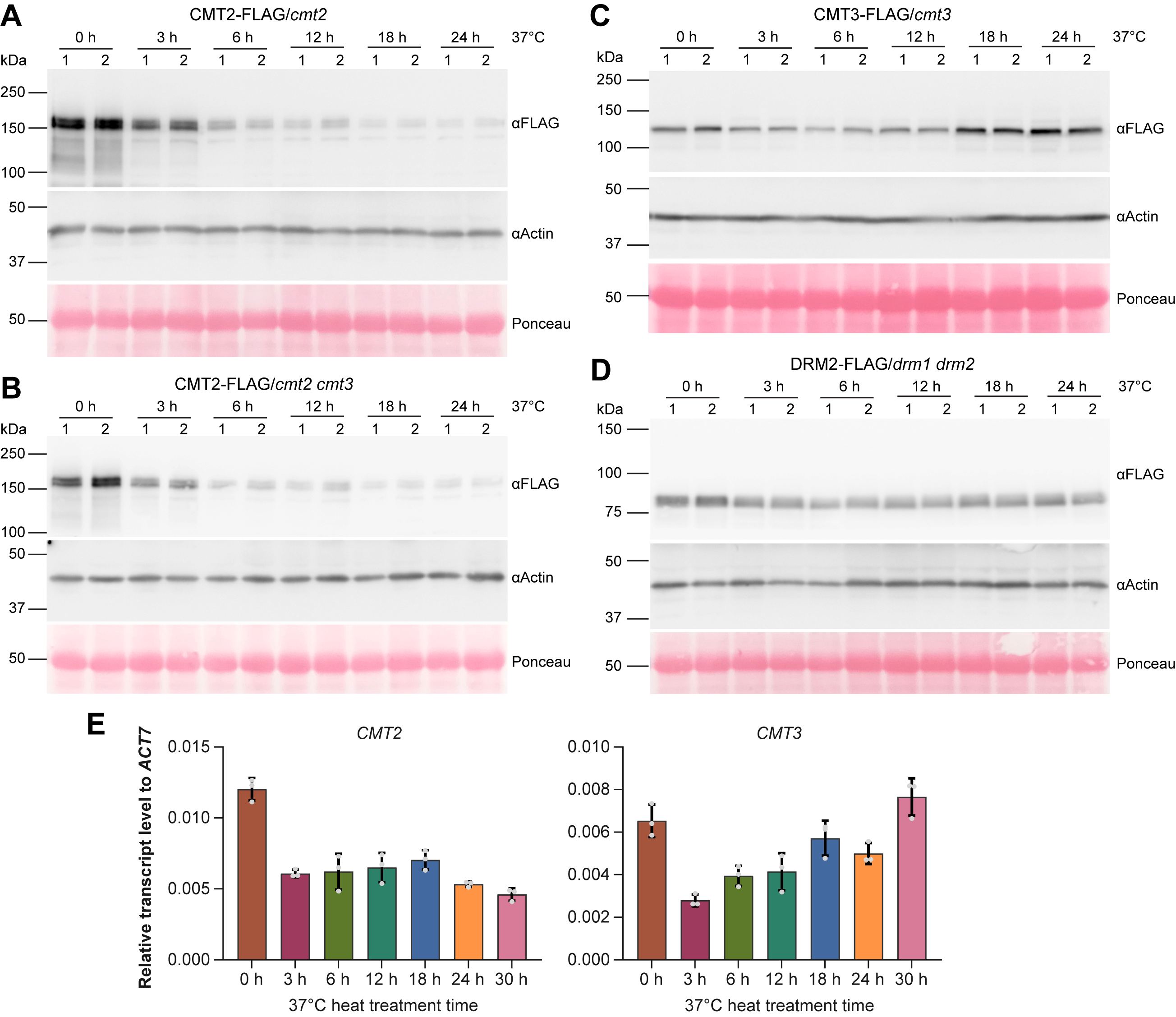

Supplement: S8 Fig — (A and B) The protein levels of CMT2-FLAG in cmt2 (A) and cmt2 cmt3 (B) backgrounds after time-course heat treatments. (C and D) The protein levels of CMT3-FLAG (C) and DRM2-FLAG (D) after time-course heat treatments. 7-d-old seedlings were subjected to 37°C heat treatment for indicated time and two biological replicates were present at each time point. Actin and ponceau staining serve as loading controls. (E) Relative transcript levels of CMT2 and CMT3 in Col-0 plants treated with 37°C heat for indicated time. The transcript levels were normalized to ACT7. Data are mean ± SD from three technical replicates at each time point. (TIF) [file pgen.1009710.s008.tif]

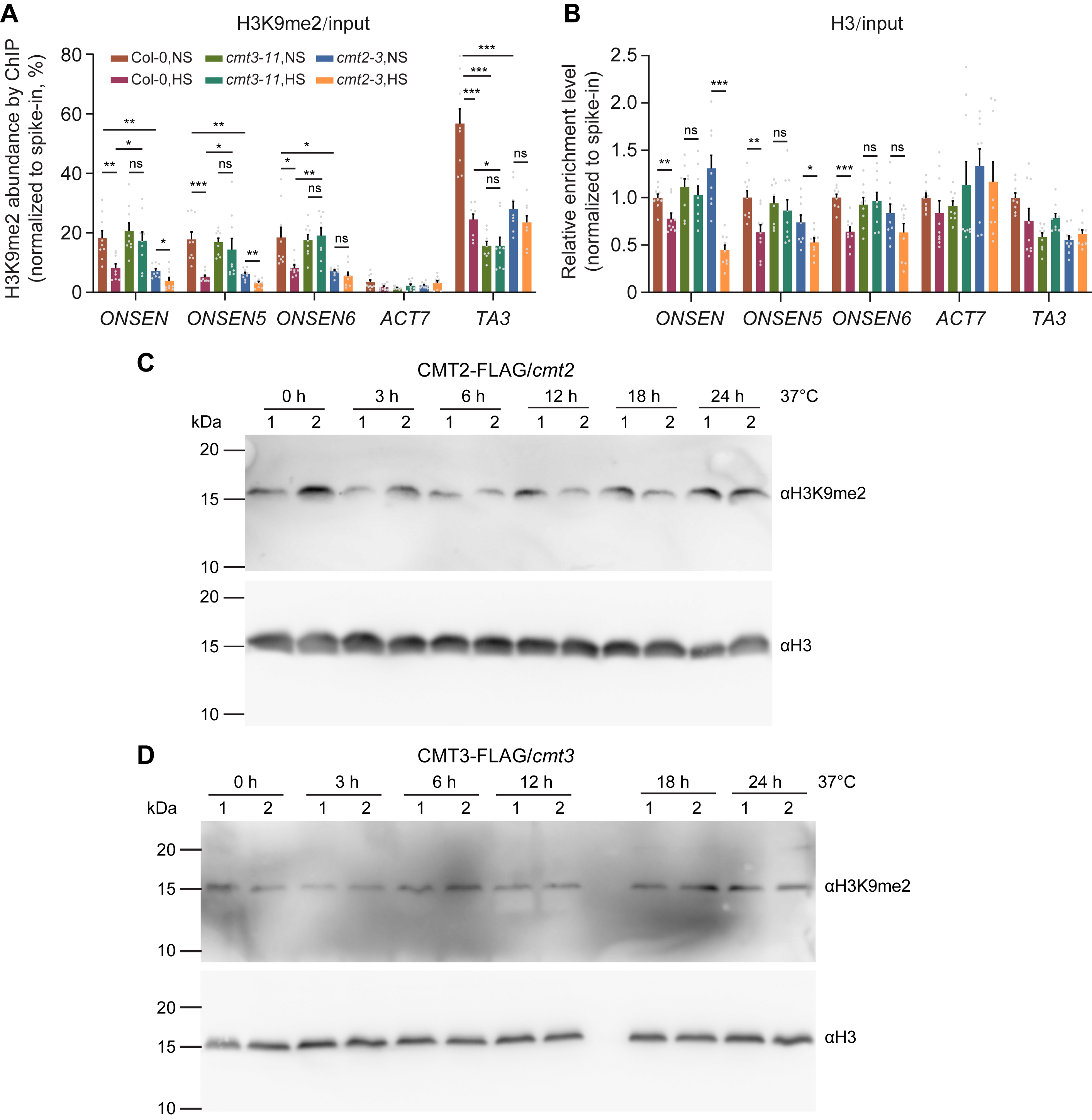

Supplement: S9 Fig — (A and B) ChIP-qPCR showing H3K9me2 (A) and H3 (B) abundance at ONSEN in Col-0, cmt3, and cmt2 under NS and HS. ChIP samples were first normalized to input, and then to the respective spike-in of human chromatin. ACT7 served as a negative control. TA3 was used as a positive control. All bars represent mean + SEM from three biological replicates with all technical replicates shown. Student’s t-test, *P < 0.05; **P < 0.01; ***P < 0.001. ns indicates not significant. (C and D) Immunoblots of H3 and H3K9me2 in CMT2-FLAG/cmt2 (A) and CMT3-FLAG/cmt3 (B) plants. 7-d-old seedlings were subjected to 37°C treatment for indicated time points. Two biological replicates were present at each time point. (TIF) [file pgen.1009710.s009.tif]

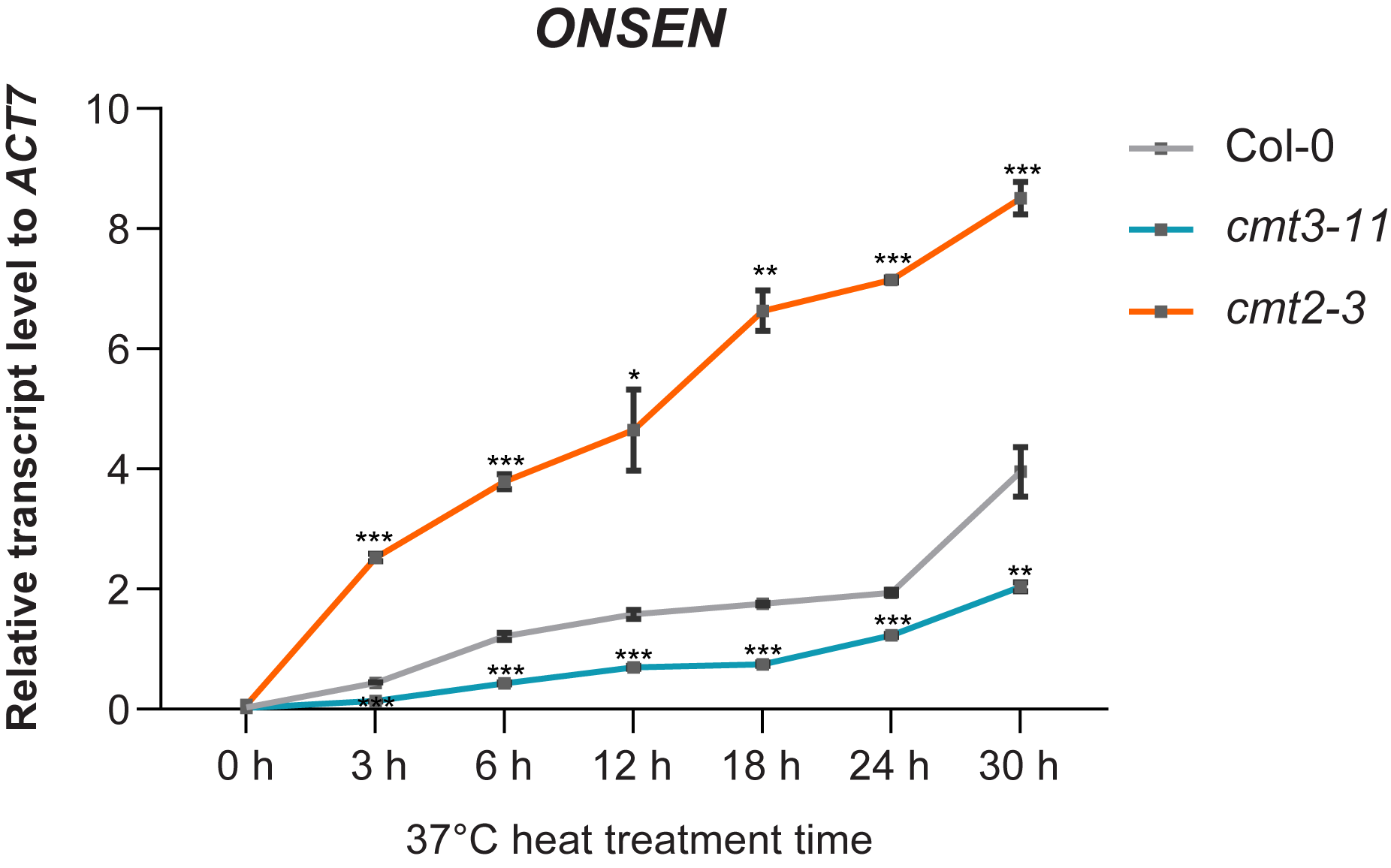

Supplement: S10 Fig — Relative transcript level of ONSEN in plants treated with 37°C heat for indicated time. The transcript levels were relative level of ONSEN to ACT7. 7-d-old seedlings were subjected to 37°C treatment for indicated time. For each time point, data are mean ± SD from three technical replicates. Student’s t-tests were performed against Col-0 at the same time point. *P < 0.05; **P < 0.01; ***P < 0.001. (TIF) [file pgen.1009710.s010.tif]
