# Supplementary material for: DNA methyltransferase CHROMOMETHYLASE3 prevents ONSEN transposon silencing under heat stress
Source: PLoS Genet. 2021 Aug 19;17(8):e1009710. doi: 10.1371/journal.pgen.1009710 (PMC8376061; doi:10.1371/journal.pgen.1009710)
Supplement: Attachment — Submitted filename: Response to reviewers_20210524.pdf [file pgen.1009710.s015.pdf]

Point-to-Point Responses to referees

We thank editor and reviewers for the constructive and encouraging comments. We have significantly improved this manuscript by performing additional experiments and more bioinformatic analysis. Below are our point-to-point responses. The comments are in black and our responses are in blue.

Reviewer #1:
The study by Nozawa, Chen et al describes an interesting mechanism whereby a DNA methyltransferase thought to be involved in silencing of repeats/transposons is actually needed to prevent silencing. This is an interesting discovery that will impact the way people think about how these pathways function in plant genomes. This study shows that CMT3 is required to prevent ectopic H3K9me2 accumulating at *ONSEN* upon heat stress. The ectopic H3K9me2 recruits CMT2 to bind and methylate DNA thereby reducing *ONSEN* activation compared to its activation upon heat stress in wild type. This study nicely incorporates genetics, WGBS and ChIP data to convincingly show that *ONSEN* requires CMT3 for heat stress induced transcription.

Overall, this study will make a nice contribution to the field. However, I would like the authors to consider a different model that in my opinion better describes the data and fits with recently published studies in the field that are considering the role of heterochromatin homeostasis.

The interpretation of the data is that CMT2 methylating CHH is what leads to silencing in *cmt3* mutants. There is no doubt CMT2 is required for this process as nicely shown by the genetic data. This leads the authors to conclude that the function of CMT3 is to prevent binding of CMT2 thereby allowing transcriptional activation upon heat stress. Their data nicely show that CMT3 is a better binder of H3K9me2 regions compared to CMT2, which is what leads to this model. It is certainly a possibility for what is happening.

Another model that should be presented, which I think better describes the data, is the increase in H3K9me2 that occurs at ONSEN in *cmt3* is what leads to the dampened transcriptional response. CMT2 binding and methylating CHH is important, but it is secondary to greater amounts of H3K9me2. More H3K9me2 equals more CMT2 and CHH as there is no CMT3 to compete with. I don't believe CMT3's function is to compete for binding with CMT2 to enable activation upon heat stress (although it's certainly one possibility). This is not to say H3K9me2 alone is sufficient for the dampened result, as it would need CMT2 to help maintain a feedback loop. Loss of either would result in greater transcription upon heat stress (as *cmt2/3* mutants show in this study).

The observed situation at *ONSEN* under heat stress fits models whereby *cmt3* mutants leads to H3K9me2 redistribution and an imbalance in heterochromatin homeostasis. Recent work by Zhang Y, et al in *PNAS* present evidence for the idea of heterochromatin homeostasis. This model can also be concluded using the data presented in this study. Therefore, I think the authors should include two potential models 1) the model presented in the study and 2) a model whereby increased H3K9me2 due to redistribution of H3K9m2 as a result of loss of CMT3.
Response: We appreciate this reviewer's enthusiastic comments and suggestion on an alternative model. We have performed more ChIP-qPCR analysis and our new data indeed showed an ectopic accumulation of H3K9me2 in *cmt3* mutant (new Fig 5F), suggesting a collaborative role of H3K9me2 and CMT2-mediated CHH methylation in preventing heat-induced *ONSEN* activation. As suggested, we have generated a new working model in Fig 6.

Minor comments:
1. It states multiple times in the abstract and the main text that *cmt3* is required for heat induced expression of *ONSEN*. As show in Figure 1, *ONSEN* activation occurs in *cmt3* mutants, it's just not as strong. Therefore CMT3 isn't require, yet it's important for a full transcriptional response.
Response: Thanks for these comments. We agree with this reviewer and have extensively edited the abstract and main text to more accurately describe the function of CMT3.

2. How many CHG sites are in the ONSEN locus, specifically where CHH is observed. Is it possible the low CHG is because there are few sites to actually methylate?
Response: We calculated the total number of C sites on both strands of the eight *ONSEN* copies

and identified 2058 CG, 719 CHG, and 12934 CHH sites. We found a significant lower proportion of CHG and higher proportion of CHH at *ONSEN* compared to all TEs. The low level CHG methylation is indeed likely due to the fact that there are few available sites to methylate. We have included this new data in Fig S5C of the revised manuscript.

3. It was established years ago by Guoll and Baulcombe that CMT2 preferentially catalyzes CWA methylation in Arabidopsis. The study would benefit from specifically measuring CWA levels as opposed to CHH. This study should also be cited as part of this manuscript.
Response: As suggested, we have analyzed the methylation of CWA and presented the new data in Fig 3D and Fig 5D of the revised manuscript. We have also cited the suggested paper (line 147).

4. It is presented numerous times that CMT3 is activating transcription. I do not agree with this conclusion. This conclusion is made based on a *cmt3* mutant. CMT3 is preventing silencing, which is not the same as activating transcription even though both result in the same amount of transcript abundance.
Response: We appreciate this comment and agree with this reviewer that "preventing silencing" more accurately describes the function of CMT3. Thus, we have extensively edited the abstract and main text in the revised manuscript.

5. Line 104 = do not equate chromatin decondensation with "open chromatin". Open chromatin refers to accessible chromatin regions bound by transcription factors that are typically 400-600bp in size. This is unrelated to the observed structural decondensation in this study.
Response: As suggested, we have changed the description of "open chromatin" to "decondensation of chromatin".

6. ChIP seq would be more compelling to show CMT2/CTM3 binding if possible, although this is not required.
Response: We have initially considered to perform the ChIP-seq to profile the genome-wide CMT2 and CMT3 binding, but ultimately didn't pursue due to the following aspects. First, this manuscript mainly focuses on *ONSEN.* The extensive genomic analysis of thousands of CMT2/CMT3 targets will greatly distract the readers' attention. Second, our new data discovered an unexpected CMT2 degradation upon the heat treatment (new Fig 4D and Fig S8A-B). While it is a very interesting observation, we don't know the underlying mechanism, which will be a great topic for future study. We plan to perform more experiments such as a series of CMT2 ChIP-seq before and under different time points of heat treatment to examine whether different genomic regions/features have different sensitivity to the reduced CMT2 protein level. As an alternative solution, we have performed more ChIP-qPCR analysis and include this new data in Fig 4B-C.

Reviewer #2:
In this manuscript entitled "DNA methyltransferase CHROMOMETHYLASE3 is required for *ONSEN* transposon activation in heat stress" the authors investigate the consequences of mutations in CMT2 and CMT3 for the transcriptional activation of the COPIA element ONSEN in response to heat. CMT2 and CMT3 are involved in the maintenance of DNA methylation in heterochromatic regions. Unexpectedly, the authors observed a reduction of ONSEN transcript level in the cmt3 mutant. The authors propose that this is caused by an increase of CMT2-mediated CHH methylation and H3K9me2 at ONSEN. The authors propose that in wild type, CMT3 inhibits CMT2 binding and CHH methylation of ONSEN, and by this function promotes ONSEN transcription under heat.
My major criticism on this manuscript is that the conclusions are based on rather minor changes of DNA methylation and H3K9me2 that have been mainly obtained under non-stressed conditions. However, since the model is based on changes happening under heat stress, changes of epigenetic marks should be investigated under heat stress conditions.

Major comments:
1. The authors propose that CMT3 directly binds to *ONSEN* and impairs binding of CMT2. However, there is only little CHGm on ONSEN, which seems at odds with this model. It is possible that CMT3 has a larger effect on *ONSEN* under heat, which could explain this discrepancy. However, data shown in Figure 3D are not convincing and it is also unclear how they were

generated. Additional evidence would be required to support the proposed model.
Response: We think that CMT3 binds to *ONSEN* chromatin via its H3K9me2 binding capacity. As requested by reviewer 1, we have calculated the total number of C sites on both strands of the eight *ONSEN* copies and identified very few CHG sites (new Fig S5C). The data in Fig 3D (now Fig 3E) are bisulfite Sanger sequencing results by amplifying the ORFs of two *ONSENs* from bisulfite converted DNA from indicated plants with or without heat treatment. As suggested, we have generated several new data and showed an ectopic accumulation of H3K9me2 in *cmt3* mutant (new Fig 5F) accompanied with increased CHH methylation. These results suggest a collaborative role of H3K9me2 and CMT2-mediated CHH methylation in preventing heat-induced *ONSEN* activation. Based on these data, we have generated a new working model in Fig 6.

2. Figure 3B and 5B: The authors propose that CHH methylation in ONSEN is increased in cmt3. This is not obvious based on the data presented. Also the metagene plots do not allow to judge quantitative changes. Boxplots would be more suitable to judge whether there are statistically significant changes of CHHm in ONSEN. This would need to be done under non-stress and stress conditions.
Response: We have remade the track data and updated the snapshots. The new Fig 3B and 5B showed notable CHH methylation increases in *cmt3*. As suggested, we have also generated boxplots for both CHH and CWA (a preferred target of CMT2) and performed more quantitative analyses. These new data are now included in Fig 3D and 5D. As suggested, we have included the bisulfite Sanger-sequencing of the ORFs of two copies of *ONSEN* under both non-stress and stress conditions (Fig 3E). We didn't observe a significant DNA methylation change. This is expected as short heat treatment time (24h) is likely not enough time for cells to divide and generate visible methylation changes.

3. Figure 5E: The data would need to be normalized to H3 rather than INPUT. Since heat stress causes chromatin decondensation, any changes in H3K9me2 levels could rather be a consequence of nucleosome density changes than changes in H3K9me2 per nucleosome.
Response: We normalized the H3K9me2 to input by following a similar study (Pecinka et al., The Plant Cell) on H3K9me2 and H3K4me3 ChIP-qPCR under heat stress. As an alternative measurement, we used western blot to examine the overall H3 abundance under the different time points of heat stress (0, 3, 6, 12, 18, and 24 hours). Our new data showed similar H3 levels across the entire time-course experiments (Fig S9).

4. The authors propose that CMT3 is not sufficient for ONSEN repression, whereas CMT2 is. A discussion on the possible differences of CMT3 and CMT2 to mediate repression is required.
Response: CMT3 and CMT2 are paralogues with different preference on substrate context. CMT3 prefers CHG, while CMT2 prefers CHH, especially CWA. Our new data showed a low number of CHG sites and also CHG methylation at *ONSEN*, while both the number and methylation level of CHH are high (Fig S5C). Thus in the case of *ONSEN*, CHH methylation mediated by CMT2 plays a more important role for repression. We have included this point in the text and discussion.

5. Different qPCR experiments were performed with different controls to normalize; is there any justification?
Response: We apologized for the confusion. For qPCR experiments, different controls used for normalization is because the experiments were performed in two labs independently. In both cases, the results are consistent.

Minor comments:
6. The proposed role of CMT3 in the activation of transposable elements should be revised; the authors implicate that CMT3 has an active role in TE activation; but its role is rather indirect in preventing repression. This should be clarified to avoid misconceptions.
Response: We appreciated these comments. As suggested, we have changed the description to avoid misconceptions.

7. L153 "negatively correlating with the increased CHH methylation level": Rephrase to "consistent with decreased CHH methylation".
Response: We have revised accordingly.

8. L222-224 rephrase this sentence; it implicates that CMT2 is required for ONSEN activation.
Response: We have revised accordingly.

9. Fig1A: The captions indicate two biological replicates, while the barplot shows three datapoints for some groups.
Response: We have corrected the errors and rewritten the caption.

10. Fig2 and Fig 4A,B: Single data points should be added to the barplots as done for Fig 1A
Response: We have added the data points to all bar plots in the revised manuscript.

11. Fig 5D: The statistical comparisons need to be specified, especially the double asterisks over the bar of suvh456 and cmt3 suvh456, do they refer to the comparison to Col-0?
Response: We have added the description of statistical comparisons accordingly.

12. Line 299-306: References for all mutants should be included.
Response: We have added the references accordingly.

Reviewer #3:
The authors show an absence of ONSEN upregulation upon heat shock in cmt3 mutant, together with an increase of H3K9me2 compared to WT. They provide data showing that both CM2 and CMT3 bind an ONSEN locus, leading to a model of ONSEN regulation. The findings are interesting although they are restricted to ONSEN family. Why is it so? The authors mention in the discussion that other TEs could be regulated this way but they didn't find any in their RNAseq data.
Response: We have re-analyzed published BS-seq data (Stroud et al., 2014) by searching *cmt3*-CHH hyper DMRs against Col-0 and then overlapping the DMRs with TEs. All 8 *ONSENs* were identified by this analysis, further confirming our conclusion. Besides *ONSENs*, we also found many other TEs particularly of DNA/MuDR, DNA/HAT, and RC/Helitron showed increased CHH methylation in *cmt3*. Further analysis demonstrated that these TEs are featured with lower CHG and higher CHH sites compared with the average of all TE, like *ONSEN*. We have included these new data in Fig S5 and S2 Data of the revised manuscript.

The manuscript is a bit confusing as most experiments are done on one *ONSEN* locus (what about the others?) and with few replicates. The manuscript would benefit from having data on transgenic lines (Fig 4) and mutants (Fig 5C, D) under stress conditions.
Response: We focus on *ONSENs* because they are the most upregulated TE by heat stress, mainly due to the heat-shock elements in the LTR regions (Ito et al., 2011, Nature; Cavrak et al., 2014, PLoS Genetics). Besides *ONSEN*, there are other TEs being upregulated by heat (Pietzenuk et al., 2016, Genome Biology; Sun et al., 2020, Nature Communications), probably due to chromatin decondensation, 3D structure change, and H3K9me2 abundance change. As suggested, we have included more data using transgenic lines and mutant under both non-stress and stress conditions in the revised manuscript.

In the discussion part the authors mention that the HS is too short to induce DNA methylation changes, however H3K9me2 marks are drastically reduced in WT (Figure 5E). Did the authors anchored the primers for ChIP in the flanking borders of ONSEN? The very high level of eccDNA (not bearing histone marks) could affect the measure.
Response: We appreciate these comments. The ChIP-qPCR primers for *ONSEN* locate within the ORF of *ONSEN,* thus it is indeed possibly in eccDNA. To solve this problem, we designed two other pairs of primers specific for *ONSEN5* and *ONSEN6*, flanking the border and with H3K9me2 ChIP signal from published ChIP-seq data (below). We checked the DNA content of *ONSEN* in the input samples of H3K9me2-ChIP and indeed found more *ONSEN* DNA using the old primers, but not *ONSEN5* and *ONSEN6*. We have updated the ChIP-qPCR results using more primers and included these new data in Fig 5F of the revised manuscript.

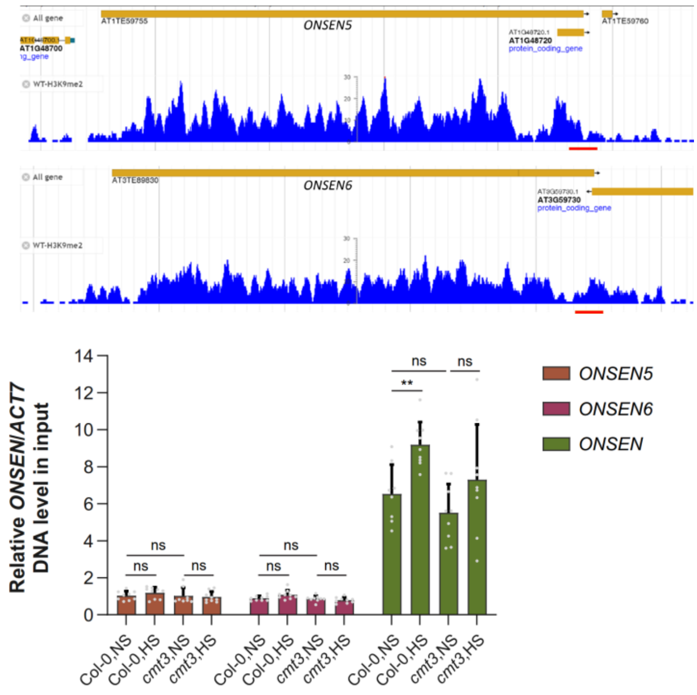

Fig 1A, Fig 2, Fig 5D,E: I wonder if it is feasible to calculate SD with only two biological replicates.
Response: We have added more data points and corrected them in the revised manuscript.

Fig 4: why were these experiments done without heat shock?
Response: We have added more data under heat stress in the revised manuscript.

Line 36: class II TEs do change copy number when they jump before replication that's why they are abundant in genomes.
Response: We have changed accordingly.

Line 43: evolutionary new insertions, do you mean 'recent insertions'?
Response: We have changed accordingly.

Line 114-115: correct typo
Response: We have corrected the typo accordingly.

Line 135: showed a greatly increase, please correct
Response: We have corrected accordingly.

Line 136: Sanger not sanger
Response: We have corrected accordingly.

Line 174: please correct the sentence
Response: We have corrected accordingly.

Line 208: a unique ONSEN, please correct to unique family
Response: We have changed accordingly.

Line 240: what is the link with the Swedish accessions?
Response: We have changed accordingly.

Line 263: the short HS treatment performed in this study is not sufficient for DNA methylation changes to occur but it is sufficient for H3K9me2 changes to occur (see Fig 5E), this should be notified here.
Response: We have changed accordingly and revised the model accordingly.

Line 276: reproduction?
Response: We have changed accordingly.

Figure 4: on this Figure the authors show that both CMT3 and CMT2 bind an ONSEN locus in WT, without HS. What happens during the HS?
Response: We have added more data under heat stress in the revised manuscript.

Figure 6: the two panels on the right (cmt3 mutant) are similar but with different outcomes for transcription, the H3K9 methyltransferase activity should be shown here. Also the grey/white arrows in the middle are confusing (between WT and mutant).
Response: We have extensively modified the model in Fig 6 of the revised manuscript.
