# Supplementary material for: DNA methyltransferase CHROMOMETHYLASE3 prevents ONSEN transposon silencing under heat stress
Source: PLoS Genet. 2021 Aug 19;17(8):e1009710. doi: 10.1371/journal.pgen.1009710 (PMC8376061; doi:10.1371/journal.pgen.1009710)
Supplement: Attachment — Submitted filename: Response to reviewers_20210707_XZ.docx [file pgen.1009710.s016.docx]

**Point-to-Point Responses to Referees**

We thank editor and reviewers for the constructive and encouraging comments. We have performed additional ChIP experiments and data analysis. Below are our point-to-point responses. The comments are in black and our responses are in blue.

**Reviewer #1:**

The new manuscript is improved for clarity and will make a nice contribution to the field. My only remaining comment is to consider presenting CWA vs non-CWA in Figure 3/5 D. If this is entirely a CMT2 dependent process the CHH shown in 3d/5d will likely be mostly CWA. I think that would be good for the reader to know.

Response: As suggested, we have separated CHH methylation to CWA and non-CWA and presented these new data in revised Fig 3E and Fig 5D. Indeed, there is no or less significant (P=0.054) CHH methylation difference between Col and *cmt3-11* at non-CWA sites.

**Reviewer #2:**

In the revised version of the manuscript, the authors included new data showing CMT2 binding and H3K9me2 accumulation under heat stress. This is an important improvement. Based on their model, CMT3 prevents CMT2 binding, causing decreased ONSEN expression in cmt3 under heat stress. The data of non-heat treated cmt3 mutants are consistent with the model. However, the new data reveal that there is no detectable CMT2 binding in cmt3 under heat, which the authors explain by showing that CMT2 is unstable under heat. What remains inconsistent with the model is the fact that H3K9me2 levels on ONSEN are not increased in cmt3 under heat (Figure 5F). One possible explanation could be that the ChIP data have been normalized to INPUT, which, as pointed out before, will lead to a misinterpretation of the data since the chromatin decondenses under heat. Most likely, the level of H3K9me2 per nucleosome will not change under heat, contrary to what the data show. The Western blots are not addressing this point since nucleosome changes in response to heat include gain and loss of nucleosomes. Preferentially this point should be experimentally addressed (ChIP normalized to H3); but at least it needs to be discussed that the data need to be treated with caution since normalization to input does not account for changes in nucleosome density.

Response: We appreciate this reviewer’s comment and suggestion. In this revised manuscript, we have performed additional H3 and H3K9me2 ChIP experiments as suggested and normalized the H3K9me2 data to H3. The new data showed reduced H3K9me2 levels in Col-0 under heat stress, even though H3 is lower under heat stress. In contrast to Col-0, H3K9me2 level in *cmt3* does not reduce under heat stress (new Figs 5F and S9). The high H3K9me2 accumulation in *cmt3* is likely due to the increased CHH methylation and also the H3K9me2-CHH methylation feedback loop. Based on these new data, we have revised the model in Fig 6.

The title remains misleading; the fact that in the cmt3 mutant ONSEN is less expressed, does not mean that CMT3 is required for ONSEN expression. The correct description of the data, which should be reflected in the title, is that "CMT2 prevents activation of ONSEN under heat stress", or "CMT3 prevents silencing of ONSEN under heat stress". To propose that CMT3 activates ONSEN would require to show that by artificially targeting CMT3 to the ONSEN locus leads to ONSEN activation, which has not been tested and seems also unlikely.

The abstract should be rephrased along the same lines.

Response: We agree with this reviewer and have changed the title to “DNA methyltransferase CHROMOMETHYLASE3 prevents *ONSEN* transposon silencing under heat stress”.
